# The Medical Examination of Recruits

**Published:** 1916-02-05

**Authors:** 


### THE MEDICAL EXAMINATION OF RECRUITS.

### An Effort to Amend the Muddle.

The relations of the medical profession to the war become more and more conspicuous. The appeal is still for more doctors for the new Armies, and recently there has been some fairly free criticism of the methods adopted in the medical examination of recruits. The conditions which existed during the last week or two of the operation of Lord Derby's scheme were without question highly unsatisfactory. Indeed, they were quite beyond defence, and it was simply impossible for the medical officers to make adequate and judicial examination of the numbers presenting themselves. But to blame the doctors for this state of things is absurd. The responsibility in the last resort falls on those who put off attestation to the eleventh hour, and then overwhelmed the officials at the last moment. No one wants to blame men who have voluntarily come forward to serve the country at the sacrifice of personal interests and ambitions, but the circumstances being what they actually were there was inevitable confusion and haste. As a necessary consequence mistakes were made, and they were made in both directions. Men were accepted who have since proved quite unfit for military duties, and on the other hand some were rejected who would have made good soldiers. To search for scapegoats is an idle task, and the authorities have wisely decided to reform the system rather than to censure individual errors. Individuals have suffered inconvenience, and still more the country has lost a certain number of efficient fighting men.

### A Better System.

An important feature of the new plan is that each candidate shall be examined in a quiet room, as, indeed, anything like efficient medical examination obviously demands. Further, greater care is to be taken to instruct the doctors in the exact conditions which the Army prescribes, and at none of the examining stations are more than 200 recruits to be presented on any one day. Of great value, too, is the regulation which prescribes that the men shall be classified in regard to the duties for which they are fit. Hitherto the duty of acceptance or rejection has been too absolute, whereas quite manifestly there must be many men who, though quite unable to suffer the full rigour of military discipline, can be raised to this level by suitable training. Sir Richard Douglas Powell has drawn attention to this aspect of the question, and has proposed that men accustomed to sedentary occupations should have two or three months of graduated physical exercises before being plunged into the strenuous drill and training which are the lot of the ordinary recruit. If this suggestion were adopted it is certain that fewer men would break down in the earlier weeks of their training, and the Army would retain a number of valuable soldiers. It has to be remembered that a large proportion of those now joining the Army come from a class in which active muscular work or exercises are not cultivated, and naturally many of these are not immediately equal to the full stress involved in drill, route marching, trench digging, and so on. The doctors who stand at the gates which guard the entrance to the Army have a large responsibility, and it is right that the conditions of their work should be adapted to this responsibility. The new arrangements offer considerable improvements in this direction, and it seems probable they will enable justice to be done both to the individual and to the country. A word of recognition must be given to the determination to give to doubtful cases the benefit of an expert's opinion. This has often been urged on the War Office authorities, and these are to be congratulated on their decision to accept medical advice. It may be quite right for the soldiers to determine what qualities are necessary for efficient military service, but whether these qualities are possessed by the individual is a question which only the doctors can settle; and in given instances a very experienced judgment may be required.

### Some of the Difficulties.

It has to be remembered that the medical examination of recruits demands alertness in two different directions. The one is the recognition of defects and the other is the detection of imposture. Some of the former may be obvious enough to the medical eye, even when unsuspected by the candidate himself, but in regard to others there may be room for difference of opinion. The question has relation not to the ordinary conditions which obtain in civil life but to the very different circumstances provided by the existing war, and an error of judgment may have serious consequences. At the very lowest the acceptance of a candidate who subsequently breaks down means expense to the country, while, if the collapse occurs on active service, the man may fail his comrades at a critical moment and in any event he becomes a burden to the Army instead of a helper. Therefore, it is a serious matter to pass into the ranks a candidate in whom such possibilities exist, and to fix an absolute and well-defined standard is almost impossible. Whatever may be done in this direction there must be something left to the discretion of the individual examiner, and it is right and proper that when he is in doubt there should be an opportunity for appeal to a senior judgment. Perhaps this opportunity is specially necessary when the candidate, in order to avoid service, professes a disability for which no physical explanation can be found. It is to be hoped that there are not many men of this order, but it would be too much to hope that there are none. The point is particularly illustrated by a profession of inability to pass the sight tests. Everyone has heard of men who have managed to evade or dodge these tests in spite of very defective eyesight. But the other side of the picture is the man who calmly says he cannot read the letters on the test types. The question then is whether he cannot or whether he will not, and the answer demands a highly technical examination. All the recruits are not now volunteers, and this creates new possibilities for the medical examiner. Such an issue as the one just suggested—and there are others—makes the need for specially expert opinion an imperative one.

A feature in the new regulations which gives us special satisfaction is the opportunity given to the examining officer to register a candidate as fit for home service while marking him as unsuitable for service abroad. We ventured to advocate this at the outset of the war, and though the decision has been somewhat delayed, we welcome it as certain to prove of value.

